# Supplementary material for: Retrospective study of the immunogenicity and safety of the CoronaVac SARS-CoV-2 vaccine in people with underlying medical conditions
Source: Commun Med (Lond). 2022 Nov 25;2:151. doi: 10.1038/s43856-022-00216-2 (PMC9700702; doi:10.1038/s43856-022-00216-2)
Supplement: Supplementary file 4 — Supplementary Information [file 43856_2022_216_MOESM4_ESM.pdf]

Supplementary Information

Retrospective study of the immunogenicity and safety of the CoronaVac SARS-CoV-2 vaccine in people with underlying medical conditions

Chunmei Li[1, §], Hanfang Bi[1, ┼], Zhenwang Fu[2, ┼], Ao Li[1], Na Wan[1], Jun Hu[3], Fan Yang[1], Tai-Cheng Zhou[4], Yupeng Liang[5], Wei Su[1], Tianpei Shi[1,6,7], Mei Yang[1], Rong Wang[1,6,7], Wanting Qin[1,6,7], Xuanjing Yu[1,6,7], Hong-Yi Zheng[8], Zumi Zhou[1], Yong-Tang Zheng[8], Jia Wei [4], Gang Zeng[5, §], Zijie Zhang[1,4, §], the Precise-CoVaccine study group*.

[1] State Key Laboratory for Conservation and Utilization of Bio-resources and School of Life Sciences, Yunnan University, Kunming, Yunnan 650091, China.
[2] Hainan Center for Disease Control and Prevention; Hainan, China.
[3] Outpatient department, The Affiliated Hospital of Yunnan University, Yunnan University, Kunming, Yunnan 650091, China.
[4] Central Lab and Liver Disease Research Center, The Affiliated Hospital of Yunnan University, Yunnan University, Kunming, Yunnan 650091, China.
[5] Sinovac Biotech Co. Ltd., Beijing, China.
[6] State Key Laboratory of Genetic Resources and Evolution, and Yunnan Laboratory of Molecular Biology of Domestic Animals, Kunming Institute of Zoology, Chinese Academy of Sciences, Beijing, 100049, China.
[7] Kunming 650223 and College of Life Science, University of Chinese Academy of Sciences, Beijing, 100049, China.
[8] Key Laboratory of Animal Models and Human Disease Mechanisms of Chinese Academy of Sciences, KIZ-CUHK Joint Laboratory of Bioresources and Molecular Research in Common Diseases, Kunming Institute of Zoology, Chinese Academy of Sciences, Kunming, Yunnan 650223, China.
┼ These authors contributed equally.

Supplementary Table 1. Antibody used for surface staining in AIM assay.

Marker-Fluorophore	Clone	Vendor	Catalog
CD3-PerCP/Cyanine5.5	OKT3	Biolegend	317336
CD4-FITC	A161A1	Biolegend	357406
CD8-APC/Cyanine7	RFT-8	Invitrogen	344714
CD69-PE/Dazzle	FN50	Biolegend	310942
CD137-APC	4B4-1	Biolegend	309810
CD134-PE/Cyanine7	Ber-ACT35 (ACT35)	Biolegend	350012
Fixable Viability Dye eFluor-506	N/A	Invitrogen	65-0866-14

Supplementary Table 2. Incidence of adverse events after vaccination in health control and comorbidities group.

Adverse events	Health (N=229)		Comorbidities (N=740)		Total (N=969)		p-value [1]
	Cases	n[2] (%)	Cases	n[2] (%)	Cases	n[2] (%)	Health vs. Comorbidities
Any adverse reactions	39	32 (13.97)	322	150 (20.27)	361	182 (18.78)	0.0334
Systemic reactions	15	13 (5.68)	161	73 (9.86)	176	86 (8.88)	0.0618
Local reactions	24	22 (9.61)	161	102 (13.78)	185	124 (12.80)	0.1129
First dose	18	16 (6.99)	158	97 (13.11)	176	113 (11.66)	0.0129
Second dose	21	19 (8.30)	164	99 (13.38)	185	118 (12.18)	0.0487

[1] The p-value was calculated by Fisher's exact test.
[2] Number of participants reporting at least 1 occurrence of the specified event category.

Supplementary Table 3. Incidence of adverse events after any vaccination dose for all participants stratified by severity of adverse events.

Adverse events	Grade 1	Grade 2	Grade 3	Total
Any adverse events	206 (16.3%)	19 (1.4%)	4 (0.3%)	229 (18.1%)
Local reactions	158 (12.5%)	5 (0.4%)	0 (0%)	163 (12.9%)
Pain	147 (11.6%)	4 (0.3%)	0 (0%)	151 (11.9%)
Induration	9 (0.7%)	0 (0%)	0 (0%)	9 (0.7%)
Swelling	14 (1.1%)	0 (0%)	0 (0%)	14 (1.1%)
Redness	3 (0.2%)	0 (0%)	0 (0%)	3 (0.2%)
Rash	3 (0.2%)	0 (0%)	0 (0%)	3 (0.2%)
Itching	8 (0.6%)	1 (0.1%)	0 (0%)	9 (0.7%)
Systemic reactions	77 (6%)	15 (1.2%)	4 (0.3%)	96 (7.6%)
Acute allergy	3 (0.2%)	2 (0.2%)	2 (0.2%)	7 (0.6%)
Skin & mucosa abnormalities	4 (0.3%)	0 (0%)	1 (0.1%)	5 (0.4%)
Diarrhoea	4 (0.3%)	0 (0%)	0 (0%)	4 (0.3%)
Anorexia	5 (0.4%)	2 (0.2%)	0 (0%)	7 (0.6%)
Vomiting	3 (0.2%)	0 (0%)	0 (0%)	3 (0.2%)
Nausea	6 (0.5%)	1 (0.1%)	0 (0%)	7 (0.6%)
Muscle pain	16 (1.3%)	3 (0.2%)	0 (0%)	19 (1.4%)
Headache	11 (0.9%)	2 (0.2%)	0 (0%)	13 (1.0%)
Cough	8 (0.6%)	0 (0%)	0 (0%)	8 (0.6%)
Fatigue	57 (4.5%)	8 (0.6%)	0 (0%)	65 (5.1%)
Fever	3 (0.2%)	0 (0%)	1 (0.1%)	4 (0.%)

Supplementary Table 4. Incidence of adverse events after vaccination in health control and different disease groups in the adults (40-59 years old) and seniors (≥60 years old) cohort.

	Healthy (N=229)		Hypertension (N=232)		Total (N=461)		p-value	
	40~59 years old (N=121)	≥60 years old (N=108)	40~59 years old(N=101)	≥60 years old (N=131)	40~59 years old(N=222)	≥60 years old(N=239)	40~59 years old	≥60 years old
Total adverse events	20 (17%)	12 (11%)	29 (29%)	17 (13%)	49 (22%)	29 (12%)	0.0349	0.6954
Local reactions	17 (14%)	5 (5%)	22 (22%)	11 (8%)	39 (18%)	16 (7%)	0.1574	0.3040
Systemic reactions	6 (5%)	7 (6%)	15 (15%)	7 (5%)	21 (9%)	14 (6%)	0.0195	0.7856
	Healthy (N=229)		CAD (N=118)		Total (N=347)		p-value	
	40~59 years old (N=121)	≥60 years old (N=108)	40~59 years old (N=31)	≥60 years old (N=87)	40~59 years old (N=152)	≥60 years old (N=195)	40~59 years old	≥60 years old
Total adverse events	20 (17%)	12 (11%)	8 (26%)	16 (18%)	28 (18%)	28 (14%)	0.2977	0.1574
Local reactions	17 (14%)	5 (5%)	6 (19%)	7 (8%)	23 (15%)	12 (6%)	0.5737	0.3778
Systemic reactions	6 (5%)	7 (6%)	4 (13%)	9 (10%)	10 (7%)	16 (8%)	0.1207	0.4326
	Healthy (N=229)		DM (N=177)		Total (N=406)		p-value	
	40~59 years old (N=121)	≥60 years old (N=108)	40~59 years old (N=71)	≥60 years old (N=106)	40~59 years old (N=192)	≥60 years old (N=214)	40~59 years old	≥60 years old
Total adverse events	20 (17%)	12 (11%)	19 (27%)	15 (14%)	39 (20%)	27 (13%)	0.0974	0.5421
Local reactions	17 (14%)	5 (5%)	13 (18%)	9 (8%)	30 (16%)	14 (7%)	0.5372	0.2819
Systemic reactions	6 (5%)	7 (6%)	9 (13%)	8 (8%)	15 (8%)	15 (7%)	0.0912	0.7950
	Healthy (N=229)		CRD (N=94)		Total (N=323)		p-value	
	40~59 years old (N=121)	≥60 years old (N=108)	40~59 years old (N=31)	≥60 years old (N=63)	40~59 years old (N=222)	≥60 years old (N=239)	40~59 years old	≥60 years old
Total adverse events	20 (17%)	12 (11%)	8 (26%)	14 (22%)	49 (22%)	29 (12%)	0.2977	0.0758
Local reactions	17 (14%)	5 (5%)	7 (23%)	10 (16%)	39 (18%)	16 (7%)	0.2721	0.0219
Systemic reactions	6 (5%)	7 (6%)	3 (10%)	6 (10%)	21 (9%)	14 (6%)	0.3893	0.5533
	Healthy (N=229)		Cancer (N=88)		Total (N=317)		p-value	
	40~59 years old (N=121)	≥60 years old (N=108)	40~59 years old (N=47)	≥60 years old (N=41)	40~59 years old (N=168)	≥60 years old (N=149)	40~59 years old	≥60 years old
Total adverse events	20 (17%)	12 (11%)	14 (30%)	5 (12%)	34 (20%)	17 (11%)	0.0853	1.0000
Local reactions	17 (14%)	5 (5%)	10 (21%)	2 (5%)	27 (16%)	7 (5%)	0.2520	1.0000
Systemic reactions	6 (5%)	7 (6%)	8 (17%)	3 (7%)	14 (8%)	10 (7%)	0.0244	1.0000
	Healthy (N=229)		Obesity (N=31)		Total (N=260)		p-value	
	40~59 years old (N=121)	≥60 years old (N=108)	40~59 years old (N=16)	≥60 years old (N=15)	40~59 years old (N=137)	≥60 years old (N=123)	40~59 years old	≥60 years old
Total adverse events	20 (17%)	12 (11%)	3 (19%)	2 (13%)	23 (17%)	14 (11%)	0.7328	0.6799
Local reactions	17 (14%)	5 (5%)	3 (19%)	2 (13%)	20 (15%)	7 (6%)	0.7048	0.2033
Systemic reactions	6 (5%)	7 (6%)	1 (6%)	0 (0%)	7 (5%)	7 (6%)	0.5895	0.5959

Supplementary Table 5. Seroconversion rate and geometric mean titer (GMT) in the health control and different disease groups in the adults (40-59 years old) cohort.

		Healthy	CAD	Hypertension	Diabetes	CRD	Obesity	Cancer
Day 14-28	Seroconversion (%)	86.00	97.00	93.00	92.00	96.00	100.00	100.00
	95% CI	(0.79,0.92)	(0.83,1.00)	(0.86,0.98)	(0.83,0.97)	(0.81,1.00)	(0.83,1.00)	(0.94,1.00)
	p-value	-	0.2000	0.1100	0.2400	0.2000	0.2200	0.0064
	GMT	28.71	33.08	49.02	29.44	30.28	47.10	53.71
	95% CI	(23.31,35.37)	(24.11,45.39)	(38.73,62.03)	(22.55,38.43)	(21.76,42.14)	(29.08,76.29)	(38.84,74.28)
	p-value	-	0.4500	0.0009	0.8800	0.7800	0.0600	0.0016
Day 90	Seroconversion (%)	53.00	58.00	65.00	51.00	62.00	71.00	72.00
	95% CI	(0.43,0.63)	(0.33,0.80)	(0.53,0.76)	(0.38,0.64)	(0.41,0.81)	(0.42,0.92)	(0.55,0.86)
	p-value	-	0.8000	0.1600	0.7500	0.5000	0.2600	0.0800
	GMT	7.70	6.89	9.97	7.31	9.25	9.17	12.21
	95% CI	(6.45,9.18)	(5.00,9.51)	(8.23,12.07)	(5.81,9.21)	(6.13,13.95)	(5.92,14.19)	(8.42,17.69)
	P-value	-	0.5400	0.0500	0.7300	0.4100	0.4400	0.0300
Day 180	Seroconversion (%)	49.00	59.00	57.00	41.00	52.00	50.00	81.00
	95% CI	(0.39,0.59)	(0.33,0.82)	(0.44,0.69)	(0.29,0.55)	(0.30,0.74)	(0.23,0.77)	(0.64,0.93)
	p-value	-	0.6000	0.3400	0.4100	0.8100	1.0000	0.0017
	GMT	8.05	8.22	8.55	6.35	8.91	6.35	15.92
	95% CI	6.57,9.86	(5.79,11.69)	(6.46,11.32)	(5.00,8.07)	(5.97,13.30)	(3.98,10.13)	(10.89,23.27)
	p-value	-	0.9100	0.7300	0.1400	0.6400	0.3300	0.0023

Supplementary Table 6. Seroconversion rate and geometric mean titer (GMT) in the health group and different disease groups in the seniors (≥60 years old) cohort.

		Healthy	CAD	Hypertension	Diabetes	CRD	Obesity	Cancer
Day 14-28	Seroconversion (%)	92.00	80.00	86.00	81.00	71.00	80.00	70.00
	95% CI	(0.85,0.96)	(0.70,0.87)	(0.79,0.91)	(0.72,0.88)	(0.59,0.82)	(0.52,0.96)	(0.54,0.83)
	p-value	-	0.0123	0.1600	0.0200	0.0004	0.1500	0.0012
	GMT	32.44	20.05	26.70	24.81	19.85	22.46	18.56
	95% CI	(26.53,39.67)	(15.29,26.30)	(22.02,32.39)	(19.30,31.88)	(14.15,27.86)	(10.04,50.25)	(12.04,28.61)
	p-value	-	0.0052	0.1700	0.1000	0.0144	0.3600	0.0200
Day 90	Seroconversion (%)	57.00	51.00	53.00	52.00	45.00	58.00	48.00
	95% CI	(0.47,0.67)	(0.38,0.64)	(0.44,0.62)	(0.41,0.63)	(0.30,0.60)	(0.28,0.85)	(0.30,0.67)
	p-value	-	0.5200	0.6800	0.5600	0.2200	1.0000	0.4200
	GMT	8.64	6.98	8.20	7.69	6.94	8.24	7.42
	95% CI	(7.32,10.20)	(5.49,8.88)	(6.97,9.65)	(6.34,9.34)	(5.34,9.01)	(4.22,16.08)	(5.31,10.36)
	p-value	-	0.1500	0.6600	0.3700	0.1600	0.8800	0.4100
Day 180	Seroconversion (%)	43.00	43.00	48.00	42.00	52.00	75.00	55.00
	95% CI	(0.33,0.53)	(0.29,0.57)	(0.39,0.58)	(0.31,0.53)	(0.37,0.68)	(0.43,0.95)	(0.36,0.73)
	p-value	-	1.0000	0.4200	1.0000	0.3600	0.0600	0.3000
	GMT	6.87	7.14	7.32	6.77	6.73	14.05	8.22
	95% CI	(5.88,8.02)	(5.41,9.43)	(6.15,8.72)	(5.47,8.37)	(5.04,8.99)	(6.26,31.55)	(5.54,12.17)
	p-value	-	0.8000	0.5900	0.9200	0.9000	0.0800	0.3900

Supplementary Table 7. Detected positive rate of SARS-CoV-2-specific CD4⁺ and CD8⁺ T cell in the health control and different disease groups.

		Healthy	CAD	Cancer	CRD	DM	Hypertension
3 months	$CD4^+$ Positive rate (%)	89.3	100.0	88.9	100.0	95.5	86.1
	95% CI	(78.1,96.0)	(86.7,100)	(51.8,99.7)	(84.7,100)	(77.2,99.9)	(70.5,95.3)
	p-value		0.181	1.000	0.325	0.666	0.746
	$CD8^+$ Positive rate (%)	60.7	38.1	66.7	61.1	72.7	38.9
	95% CI	(46.8,73.5)	(18.1,61.6)	(29.9,92.5)	(35.7,82.7)	(49.8,89.3)	(23.1,56.5)
	p-value		0.122	1.000	1.000	0.433	0.055
6 months	$CD4^+$ Positive rate (%)	97.6	100.0	81.8	90.5	96.3	100.0
	95% CI	（87.4,99.9）	（89.5,100）	（59.7,94.8）	（69.6,98.8）	（81,99.9）	（90.2,100）
	p- value		1.000	0.044	0.256	1.000	1.000
	$CD8^+$ Positive rate (%)	78.6	55.6	72.7	61.9	88.9	72.4
	95% CI	（63.2,89.7）	（35.3,74.5）	（49.8,89.3）	（38.4,81.9）	（70.8,97.6）	（52.8,87.3）
	p-value		0.061	0.757	0.229	0.342	0.582

Supplementary Table 8. SARS-CoV-2-specific $CD4^+$ and $CD8^+$ T cell fraction in the health control and different disease groups.

		Healthy	CAD	Cancer	CRD	DM	Hypertension
3 months	$CD4^+$ (%)	0.05	0.05	0.03	0.08	0.08	0.04
	IQR	（0.019, 0.106）	（0.019, 0.11）	(0.013,0.04)	(0.027,0.12)	(0.022,0.169)	(0.012,0.084)
	p-value		0.835	0.894	0.357	0.251	0.795
	$CD8^+$ (%)	0.01	0.00	0.01	0.01	0.02	0.00
	IQR	(0, 0.027)	(0, 0.01)	(0, 0.016)	(0, 0.028)	(0.001, 0.059)	(0, 0.007)
	p-value		0.087	0.676	0.573	0.134	0.058
6 months	$CD4^+$ (%)	0.07	0.07	0.08	0.08	0.06	0.04
	IQR	(0.029, 0.155)	(0.038, 0.101)	(0.012, 0.128)	(0.057, 0.109)	(0.034, 0.177)	(0.027, 0.102)
	p-value		0.764	0.462	0.631	0.439	0.817
	$CD8^+$ (%)	0.01	0.00	0.01	0.01	0.02	0.01
	IQR	(0.005, 0.039)	(0, 0.036)	(0, 0.019)	(0, 0.02)	(0.008, 0.034)	(0, 0.015)
	p-value		0.764	0.462	0.631	0.439	0.817

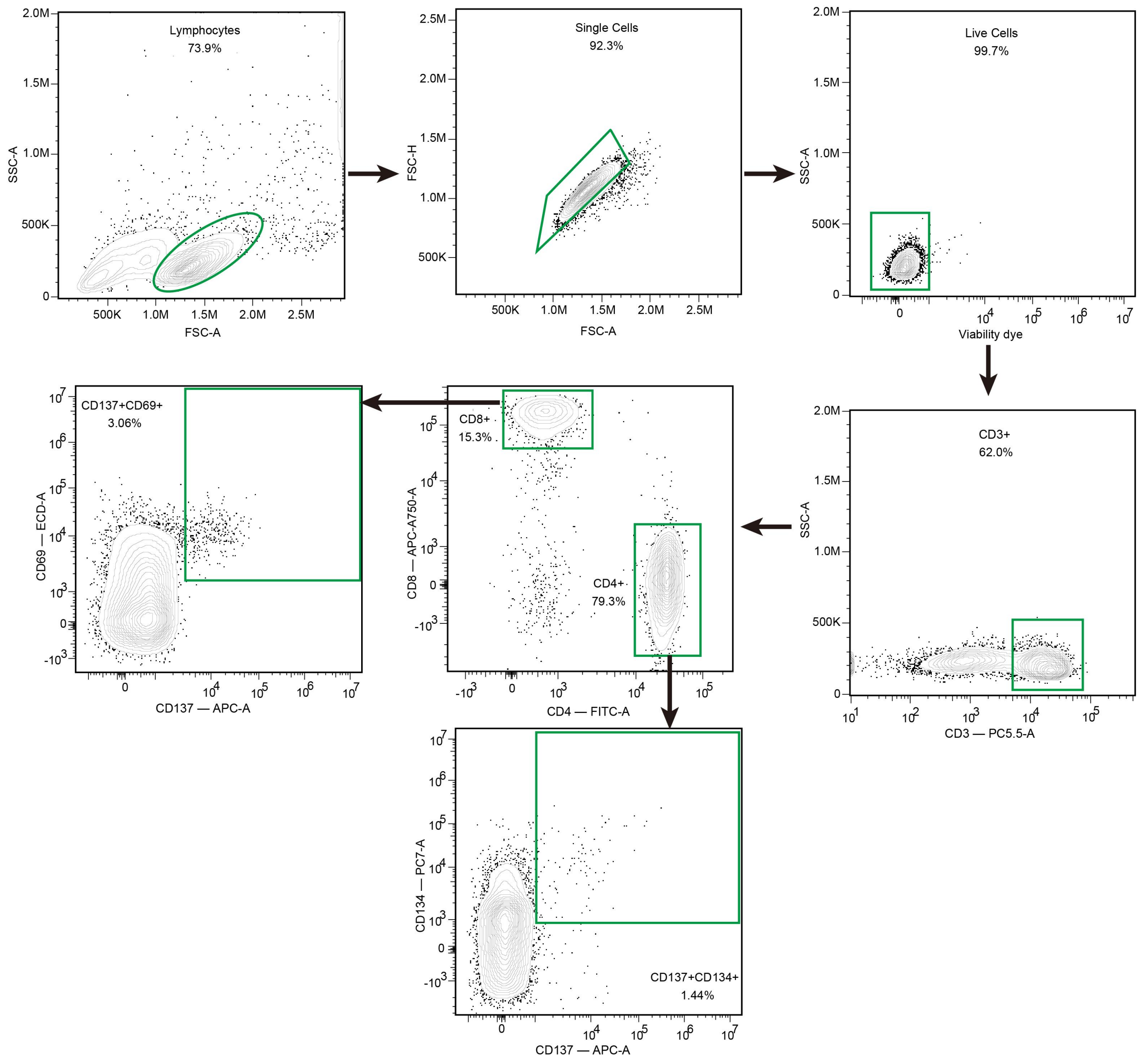

Supplementary Figure.1: The gating strategy of activation-induced cell marker (AIM) assay to quantify the SARS-CoV-2 specific T cell responses. The lymphocyte gated by the FSC/SSC; then gated the single cell by the FSC/SSC; gated $CD3^+$ for T cells; gated $CD4^+$, $OX40^+$ and $CD137^+$ for SARS-CoV-2 specific CD4 T cells; gated $CD8^+$, $CD69^+$ and $CD137^+$ for SARS-CoV-2 specific CD8 T cells;

Supplementary Note 1. Serious or severe adverse events reported during the study.

4 participants in this study had 6 cases of severe (grade 3) adverse events:

Individual 1, 60-65 years old, received the first dose of the vaccine in July 2021, and the second dose on August, 2021. Grade 3 Skin & mucosa abnormalities (mouth ulcers) was found post 14 days of both doses of vaccination and recovered within one week. In addition, there were grade 2 headache after the

first dose; and grade 2 injection-site pain, grade 1 swelling, grade 2 headache, grade 1 fatigue and grade 1 fever after the second dose. He (She) was enrolled in CRD group, with hypertension, cancer, and hyperthyroidism. His (Her) health condition is stable within 6 months before the first dose of vaccination. Since mouth ulcers are not highly associated with common adverse effects of vaccination, it is considered not associated with vaccination.

Individual 2, 80-85 years old, received the first dose of the vaccine in August 2021, and the second dose of the vaccine on September, 2021. Only a grade 3 acute allergic reaction occurred after the second dose (October), which was manifested as systemic urticaria. He (She) was enrolled in hypertension group. His (Her) blood pressure is stable within 6 months before the first dose of vaccination. Follow-up visits were conducted every half a month, and as of February 2022, he (she) still had symptoms of urticaria, which were relieved by taking drugs to treat urticaria. Considering that urticaria is an immune disease, it may be the cause of the immune system disorder in the body after vaccination, but because the onset time is more than 7 days from the vaccination, the possibility of seasonality and other factors cannot be ruled out.

Individual 3, 70-15 years old, received their first dose of the vaccine in September 2021, and the second dose of the vaccine on September, 2021. Grade 3 acute allergic reaction occurred after both doses, specifically systemic urticaria. In addition, after the first dose of vaccination, there was grade 1 swelling at the injection-site; and after the second dose, there were grade 2 fatigue. He (She) was enrolled in DM group, with hypertension. His (Her) health condition is stable within 6 months before the first dose of vaccination, usually controlled by exercise and diet in addition to drugs. Considering that urticaria is an immune disease, it may be the cause of the immune system disorder in the body after vaccination, but because the onset time is more than 7 days from the vaccination, the possibility of seasonality and other factors cannot be ruled out.

Individual 4, 46-50 years old, received their first dose of the vaccine on June, 2021 and the second dose of the vaccine on July, 2021. Grade 3 fever occurred after the second dose of vaccination, with a maximum body temperature of 38.5℃, and recovered after taking the medicine. In addition, after the first dose of vaccination, there are grade 1 injection-site pain, grade 1 swelling, grade 1 anorexia, grade 1 muscle pain, grade 1 headache, and grade 1 fatigue; and after the second dose of vaccination, there are also grade 1 muscle pain, grade 1 headache and fatigue. He (She) was enrolled in DM group, with hypertension. The fever is associated with vaccination, but this is a common adverse vaccine reaction and was cured after drug therapy.

Supplementary Note 2. Study Protocol:

This trial protocol has been provided by the authors to give readers additional information about their work.

Protocol for: Chunmei Li, Hanfang Bi, Zhenwang Fu, et al. Retrospective study of the immunogenicity and safety of the CoronaVac SARS-CoV-2 vaccine in people with underlying medical conditions. *Communications Medicine*.

Study protocol Version 1.0

CONFIDENTIAL

Title

Study on safety and immunogenicity of the CoronaVac inactivated SARS-CoV-2 vaccine (Vero cells) in a population with underlying medical conditions

Protocol Number

PRO-nCOV-4004

Sponsor: Sinovac Biotech Co.Ltd.

Research organizations: Yunnan University, Hainan CDC

Statistical Organization: Yunnan University

Investigators

Zijie Zhang PhD,

on behalf of the Precise-CoVaccine study group.

Version 1

June 18, 2021

Institution

State Key Laboratory for Conservation and Utilization of Bio-resources and School of Life Sciences, Yunnan University, Kunming, Yunnan 650091, China.

Hainan Center for Disease Control and Prevention; Hainan, China.

Central Lab and Liver Disease Research Center, The Affiliated Hospital of Yunnan University, Yunnan University, Kunming, Yunnan 650091, China.

Principal Investigator Agreement Page

I agree:

- Assume the responsibility for properly instructing this clinical trial.
- Ensure that the trial is conducted is carried out in accordance with the Trial Protocol and standard operating procedure for clinical research.
- Ensure that personnel involved in this trial are fully aware of the research product information, as well as other responsibilities and obligations in connection with the Research as specified in the Trial Protocol.
- Ensure that no changes to the trial protocol are made without review and written approval of the sponsor and the Independent Ethics Committee (IEC), unless necessary to eliminate immediate harm to subjects or as required by the registration authority (e.g., administration of the Project).
- I am fully familiar with the proper use of the vaccine as described in the trial protocol and am fully aware of other information provided by the sponsor, including but not limited to the following: the current Investigator's Brochure (IB) or equivalent document and supplementary documents to the IB (if any).
- I am familiar with and will comply with Good Clinical Practice (GCP), Guidelines for the Quality Management of Vaccine Clinical Trials (trial implementation) and all current regulatory requirements.

Investigator's Name (print):	Zijie Zhang
Investigator's Signature:	Zijie Zhang
Date of Signature:	06 21 2021

1.Background
This is a sub-study of an observational, comparative, cohort study under the umbrella program “The Precise-CoVaccine” Study.

The COVID-19 inactivated vaccine (Vero cell) which is developed by SinoVac Biotech Co., Ltd., Beijing, China (hereinafter referred to as” SinoVac”), can induce body to produce active immunity and PREVENT diseases caused by SARS-CoV-2. This vaccine has been evaluated in many randomized, double-blind, placebo-controlled studies.

The clinical trials of vaccine is authorized by National Medical Products Administration (NMPA) (certificate no: 2020L00018) on April 13, 2020. SinoVac has completed two Ⅰ/Ⅱ stage of clinical trials: one for healthy adults at the age from 18 to 59 in Suining county, Jiangsu province, China; the other for elderly people (aged 60 or more) in Renqiu city, Hebei province, China. The results show that the experimental vaccine has good safety and immunogenicity for adults and older people in 28 days after vaccination. SinoVac also has finished vaccine phase III clinical trials in Brazil for adults (18 years old or above). The results show that SinoVac vaccine has good protective efficacy. SinoVac vaccine was allowed to go public (certificate no: 2021S00156) on February 5, 2021. Now, the clinical study is carried out to provide more data to support expanding the applicability of the vaccine to the population with underlying medical conditions.

This study will be a multicenter retrospective cohort study in four different study sites (Haikou city, Wenchang city, and Qionghai city, Hainan Province; Kunming city, Yunnan Province; China), aiming to evaluate the immunogenicity and safety of CoronaVac vacancies in people with underlying medical conditions in comparison with age matched healthy control. In this study, we will recruit 1,600 participants who are 40 years of age or older, have received 2 doses of CoronaVac inactivated vaccine with 3-5 weeks of dose interval and are at the 14th-28th day after the second dose at the time of enrollment.

2. Rationale
People living with chronic disease, particularly seniors older than 60 years old, are lagging behind in the national COVID-19 vaccination campaign in China due to uncertainty of safety and effectiveness. [10, 15,16] However, this special population made up of most severe symptom and death cases among SARS-CoV-2 infected patients and should be prioritized in vaccination program.[27] Safety and immunogenicity data of COVID-19 vaccines in people with underlying medical conditions are needed to address the vaccine hesitation in this special population.

3. Study aim and hypothesis
3.1 Aim:
To evaluate the immunogenicity and safety of CoronaVac inactivated vacancies in people with underlying medical conditions in comparison with age matched healthy control.

3.2 Hypothesis:
The CoronaVac inactivated SARS-CoV-2 vaccine show no significant difference in immunogenicity and safety profiles between people with underlying medical conditions and the healthy population.

4. Study objectives

4.1 Primary objective

The primary safety endpoint is the incidence of adverse events within 14 days after the first dose and the second dose of the vaccination. The primary immunogenic endpoints are the seropositive rate and the geometric mean titers (GMTs) of neutralizing antibodies to live SARS-CoV-2 virus (wild type) 14-28 days, 90 days, and 180 days after two-dose vaccination.

4.2 Secondary objectives

The secondary immunogenic endpoint is cellular responses (as measured by AIM assay) 90 days and 180 days post two-dose vaccination.

5. Methods

5.1 Study design

We will conduct a multicenter retrospective clinical trial in four different study sites (Haikou city, Wenchang city, and Qionghai city, Hainan Province; Kunming city, Yunnan Province; China), aiming to evaluate the immunogenicity and safety of two-dose CoronaVac vaccination in people with underlying medical conditions in comparison with age matched healthy control.

We will recruit participants who have received 2 doses of CoronaVac with 3-5 weeks of dose interval and are at the 14th-28th day after the second dose at the time of enrollment. Participants are eligible if they are 40 years of age or older, healthy, or diagnosed with any of the 6 most common chronic diseases: hypertension, diabetes mellitus (DM), coronary artery disease (CAD), chronic respiratory disease (CRD), obesity, and cancer, and can understand and complete the questionnaires. The diagnose of six underlying medical conditions in our study will mainly based on the diagnosis of local hospital according to National clinical practice guidelines in China. Specifically, we will screen potential eligible participants through the CDC database of chronic diseases and vaccination. We then will recruit the volunteers who registered as healthy or with at least one of the diseases we studied by telephone interview. During the telephone visit, we will further narrow down to people who have a proof of chronic diseases diagnosis of interest from a local hospital. At the first visit, we will check their case history and asked about their treatment and disease status. People will considered as having underlying medical conditions if they were once diagnosed as one of the 5 common chronic diseases (hypertension, DM, CAD, CRD and cancer), and the disease is not in the acute phase during the recruitment, and will maintain the regular treatment during vaccination. The obesity was diagnosed as BMI≥28.0 kg/m². They will be excluded according to established criteria: had been infected by SARS-CoV-2; had received non-CoronaVac vaccine; with severe mental and neurological diseases; with any other factors unsuitable for clinical observation.

Sex matched healthy participants will recruit as the control group. Since underlying medical conditions are common in older adults (≥60 years), participants will group into adult (40-59 years old) and senior (≥60 years) subgroups to evaluate the effect of age more accurately on the immunogenicity and safety of the inactivated SARS-CoV-2 vaccine. The disease group will further divide into 6 subgroups based on the 6 common diseases we concerned.

5.2 Recruitment and consent process

- The study coordinator will contact all volunteers that have the record of healthy or disease status who have received two doses of the inactivated vaccine.
- A telephone script will be used to introduce this study and initiate the consent process.
- Questions about the study can be answered over the phone at this time.
- The study investigator(s) will be available in case of questions.
- At the first study visit, eligibility will be further reviewed.
- Consent will then be signed at local clinics, after which blood collection for serum samples will be completed.
- Venous blood will be collected for neutralizing antibody response assay at day 14-28, 90 and 180 post two-dose vaccination

5.3 Participant population

Inclusion criteria:

- The participants are 40 years of age or older, male, or female.
- The participants are healthy or diagnosed with any of the 6 most common chronic diseases: hypertension, diabetes mellitus (DM), coronary artery disease (CAD), chronic respiratory disease (CRD), obesity, and cancer.
- Two doses of CoronaVac inactivated SARS-CoV-2 vaccine are administered recently (14th-28th day after the second dose) and the interval between two doses is 3-5 weeks.
- Participants can follow the researcher's guidance to accomplish the research process.

Exclusion criteria:

- The history of SARS-CoV-2 infection.
- The history of vaccination with other types of SARS-CoV-2 vaccine other than CoronaVac.
- Severe mental and neurological disease.
- According to the researchers, any other factors that the subjects do not fit to participate in this clinical trial.

5.4 First visit

The first visit will be conducted at 14-28 days post the second-dose vaccination. We will check their case history and asked about their treatment and disease status. People will considered as having underlying medical conditions if they were once diagnosed as one of the 5 common chronic diseases (hypertension, DM, CAD, CRD and cancer), and the disease is not in the acute phase during the recruitment, and will maintain the regular treatment during vaccination. The obesity will diagnosed as BMI≥28.0 kg/m². For eligible participants, the blood samples will be collected to explore the GMT of neutralizing antibodies and cellular responses. We will record all adverse events, including serious/severe adverse events, and the changes in their chronic medical condition, occurred within 14 days after each dose.

5.5 Follow-up

Eligible participants will be followed up at 3 and 6 months after the second inactivated

vaccine dose. Blood samples will be collected at the time of 3 and 6 months after the second dose of vaccine to explore the GMT of neutralizing antibodies and cellular responses. Non-routine health-care contacts including hospitalization and the reasons for these healthcare visits will be recorded. Additionally, disease condition variation will be recorded for participants underlying medical conditions.

Type of contact	Visit 1 (Blood collection)	Visit 2 (Blood collection)	Visit 3 (Blood collection)	Passive follow-up
Time points	Day 14-28 post the second dose vaccination	3 months post vaccination	6 months post vaccination	Up to 6 months post vaccination
Eligibility review	√			
Review of signed consent form	√			
Blood draw	√	√	√	
Participant reporting of adverse events or serious adverse events	√			√
Participant reporting of their chronic medical conditions variation	√			√

5.6 Participant Confidentiality

Participants will be assigned with a unique study identifier. Initially a separate list with participant study identifier and phone number will be kept in order to contact the participant for arranging the blood collection post vaccination, which will be destroyed when study is completed. Samples will only contain study identifier and date. Samples will be kept until the completion of this study.

All records and documents pertaining to the study will be retained by the study trial site at the Hainan CDC and Affiliated Hospital of Yunnan University for up to 5 years from the completion of the study. Paper records will be stored in a secure filing cabinet in a locked office. Baseline demographic data will be collected and then stored in an electronic database. The database will be stored on the Yunnan University accessed from a password protected computer located in a secure location in the campus.

5.7 LABORATORY METHODS

5.7.1 Laboratory Methods:

Serology:

All serum samples will obtain by centrifugation of blood samples collect by a Vacutainer Rapid Serum Tube (BD) and store at -80℃ until testing. The neutralizing antibodies to live

SARS-CoV-2 will quantify using the gold standard of antibody titration: microcytopathogenic effect assay with live SARS-COV-2 virus to quantify the neutralizing antibody in all participants with serum samples. 50 μL serum is first inactivate at 56℃ for 30 minutes and serially diluted with cell culture medium in two-fold steps. The diluted serum is then incubated with equal volume of live SARS-CoV-2 (virus titers: 6.0 lgCCID 50/mL; passage: P5; GenBank number: MT407649.1) for 2 hours at 36.5℃. Vero cells are then added to the serum-virus mix and incubated at 36.5℃ for 5 days. neutralizing antibody titer is calculated by the highest dilution number of 50% protective condition.

Peripheral blood mononuclear cells (PBMCs):
For blood samples will collect at two sites (Haikou and Kunming), we will preserve whole blood using Vacutainer tubes (BD, USA) containing EDTA at room temperature for no longer than 6 hours before isolation. PBMCs are then separated by density-gradient sedimentation. In brief, whole blood is diluted by RPMI 1640 (VIVACELL, China) in a 1: 1 ratio, layered over Ficoll (STEMCELL, Canada), and then followed by a centrifugation of 30 minutes at 1455g with no brake. The PBMC buffy coat and plasma are then collected and washed by adding equal volume RPMI 1640 (VIVACELL, China). Then the liquid supernatant is removed after centrifugation for 10 minutes at 524g. Cells are aliquot by pre-cooled cryopreservation solution containing 10% DMSO (Solarbio, China) and 90% fetal bovine serum (VIVACELL, China) and transfer into cryopreservation tubes, place in a pre-cooled Mr. Frosty freezing container (Thermo Scientific). All containers are then moved into -80℃ freezer overnight before transferring to liquid nitrogen for long-term storage.

AIM assay:
SARS-CoV-2 specific mega-pool (MP) consists of commercial peptide pools (GenScript) derived from peptide scan (15 mers with 11 aa overlap) through the entire Spike glycoprotein (Protein ID: P0DTC2), Membrane protein (Protein ID: P0DTC5) and Nucleoprotein (Protein ID: P0DTC9) of the wild type of SARS-CoV-2. Cells are cultured for 24 hours in the presence of SARS-CoV-2 specific MP (1μg/mL) in 96-wells U bottom plates at 1×10^6 PBMC per well in complete RPMI containing 5% Human AB Serum (Gemini Bioproducts). Negative control is performed by adding an equimolar amount of DMSO, and phytohemagglutinin (Roche, 1 μg/mL) will included as a positive control. For the surface stain, to remove the cell culture medium, cells are washed with staining buffer (BD). Cells are then resuspended and stained with antibody cocktail (Appendix table 1) for 30 minutes at RT in the dark, washed, and resuspended by staining buffer.[25] All sample flow cytometry data is acquired on a Beckman DxFLEX.

5.8 OUTCOME MEASUREMENTS

5.8.1 Primary outcome:

The primary outcome will be the seropositive rate as measured by the neutralizing antibody titer at 14-28 days after the two-dose vaccination among people with chronic diseases and healthy controls.

5.8.2 Secondary outcome:

- The GMT of neutralizing antibody titers at 14-28 days after the two-dose vaccination among people with chronic diseases and healthy controls.

- The incidence of adverse events within 14 days after each dose of vaccination.
- The seropositive rate as measured by the neutralizing antibody titer at 90 days and 180 days after the two-dose vaccination.
- The ratio of SARS-CoV-2-specific T cells among $CD4^+$ and $CD8^+$ T cells 3 and 6 months after the second dose.
- Documented COVID-19 infection (i.e., microbiology proven by PCR from appropriate clinical specimen and compatible clinical syndrome) in the 6 months following vaccination.

6 STATISTICAL CONSIDERATIONS

For participants enrolled and completed the assays in each disease group, the age, weight, BMI will be described by mean and standard deviation; the gender and nationality will be described by the ratio among total participants.

For immunogenicity evaluation, we will use descriptive statistics (geometrical mean and 95% confidence interval [CI]) to summarize antibody levels, and the GMT of post-immunization neutralizing antibody is analyzed after logarithmic conversion, and the least square mean of GMT of post-immunization neutralizing antibody and the ratio between groups and its 95% confidence interval (95%CI) will calculated for comparison. The positive rate of neutralizing antibody after immunization is calculated for the experimental group and the control group, the bilateral 95%CI will calculate by Clopper-Pearson method, and the difference between groups will statistically tested by the Chi-square test /Fisher exact probability method. At the same time, geometric mean and 95%CI will used to statistically describe the GMT of the immune neutralizing antibodies of participants in each cohort, and logarithmic converted group T test will used to statistically compare the difference between groups.

For the T-cell response analysis, raw data will process and exported by FlowJo_v10.8.0. The detected ratio of SARS-CoV-2-specific $CD4^+$ and $CD8^+$ T cells after immunization is calculated for the experimental group and the control group, the bilateral 95%CI will calculate by Clopper-Pearson method, and the difference of positive rate between groups will statistically evaluated by the Chi-square test /Fisher exact probability method. The fraction of SARS-CoV-2-specific T cells will be described by median and interquartile range (IQR), and the difference between groups will statistically tested by linear regression analysis, considering the AIM assay experimental batch effect with a covariate.

For safety evaluation, in accordance with the protocol, systemic adverse events and local adverse events will be classified and counted. In this study, Treatment Emergent Adverse Events (TEAE) occurred after inoculation will statistically analyzed. Adverse events are expressed by counts and frequency. The number and incidence of all adverse events in each group will calculated, and the differences between groups will statistically compared using Fisher's exact test method. Descriptive statistics are made for the severity, dosage, and occurrence time of adverse events. The adverse events after each dose of inoculation will statistically analyzed. Adverse events for each dose are analyzed based on the safety population of each dose. Serious or severe adverse event reported in this study will be analyzed case by case.

7 SIGNIFICANCES OF THE STUDY

People living with chronic disease, particularly seniors older than 60 years old, made up of most severe symptom and death cases among SARS-CoV-2 infected patients.[5] However, there are a proportion of seniors aged 60 years or older remain unvaccinated, especially in China, which may further increase the mortality cases at outbreaks.[1] Most people living with chronic disease in China, particularly seniors older than 60 years old, are vaccine-hesitant (most about the inactivated SARS-CoV-2 vaccines), which has led to delays in COVID-19 vaccination. These people may worry about the safety and effectiveness of the vaccination, given those data has been rare. This study will be a systemic clinical report of the safety and immunogenicity of the CoronaVac inactivated SARS-CoV-2 vaccine on people with common chronic diseases in China. The results will provide clinical evidence for the expansion of vaccine application in people living with chronic diseases.

8 ETHICAL CONSIDERATIONS

Written informed consent will obtained from each participant before enrolment. The study protocol and informed consent form has approved by the Committee on Human Subject Research and Ethics of Yunnan University (CHSRE2021021). This study is conducted in accordance with the requirements of Good Clinical Practice of China and the International Conference on Harmonization. This study has registered with ChiCTR.org.cn, ChiCTR2200058281.

9 DEFINITIONS OF ADVERSE EVENTS

9.1 Grades of adverse events.

Participants will be required to record local adverse events (pain, induration, and redness), or systemic adverse events (e.g., allergic reactions, cough, and fever) for the first 14 days after each dose vaccination. Reported adverse events will graded according to China National Medical Products Administration guidelines. The existence of causal associations between adverse events and vaccination will be determined by the investigators.

Grading of (Local) Adverse Events and Vital Signs

	Grade 1	Grade 2	Grade 3	Grade 4
**Pain**	Having no or marginal effect on limb activity	Influencing limb activity	Influencing daily life	Loss of basic self-care ability or hospitalization
**Induration*#**	Diameter 2.5～＜5 cm or area 6.25～＜ 25 cm^2 and having no or marginal effect on daily life	Diameter 5～＜10 cm or area 25～＜ 100 cm^2 or having an effect on daily life	Diameter ≥10 cm or area ≥100 cm^2 or fester or secondary infection or phlebitis or sterile abscesses or wound drainage or having serious effect on	Abscess, exfoliative dermatitis, dermal or deep tissue necrosis

	Grade 1	Grade 2	Grade 3	Grade 4
			daily life	
**Swelling #**	Diameter 2.5～＜5 cm or area 6.25～＜ 25 cm^2 and having no or marginal effect on daily life	Diameter 5～＜10 cm or area 25～＜ 100 cm^2 or having an effect on daily life	Diameter ≥10 cm or area ≥100 cm^2 or fester or secondary infection or phlebitis or sterile abscesses or wound drainage or having serious effect on daily life	Abscess, exfoliative dermatitis, dermal or deep tissue necrosis
**Redness #**	Diameter 2.5～＜5 cm or area 6.25～＜ 25 cm^2 and having no or marginal effect on daily life	Diameter 5～＜10 cm or area 25～＜ 100 cm^2 or having an effect on daily life	Diameter ≥10 cm or area ≥100 cm^2 or fester or secondary infection or phlebitis or sterile abscesses or wound drainage or having serious effect on daily life	Abscess, exfoliative dermatitis, dermal or deep tissue necrosis
**Rash*#**	Diameter 2.5～＜5 cm or area 6.25～＜ 25 cm^2 and having no or marginal effect on daily life	Diameter 5～＜10 cm or area 25～＜ 100 cm^2 or having an effect on daily life	Diameter ≥10 cm or area ≥100 cm^2 or fester or secondary infection or phlebitis or sterile abscesses or wound drainage or having serious effect on daily life	Abscess, exfoliative dermatitis, dermal or deep tissue necrosis
**Itching**	Itching at the injection-site, relieved on its own or within 48 h after treatment	Itching at the injection-site, no remission within 48 h after treatment	Influencing daily life	NA

* Induration and rash: in addition to the grading and evaluation by measuring the diameter directly, the change of measurements should also be recorded.

# Induration, swelling, redness and rash: the maximum measured diameter or area should be used; The grading and evaluation should be based on the function grade and actual measurements and indicators with a higher grade should be chosen.

Grading of (Systemic) Adverse Events and Vital Signs

	Grade 1	Grade 2	Grade 3	Grade 4
**Acute allergic***	Local urticaria (blister), no treatment required	Local urticaria, requiring for treatment or mild angioedema, no treatment required	Extensive urticaria or angioedema requiring for treatment or mild bronchospasm	Allergic shock or life-threatening bronchospasm or laryngeal edema
**Skin & mucosa abnormalities**	Erythema, pruritus, or color changes	Diffuse rash, maculopapular rash, dryness, or desquamation	Vesicular, exudative, desquamation, or ulceration	Exfoliative dermatitis involves the mucous membranes, or erythema multiforme, or is suspected of Stevens-Johnsons syndrome
**Diarrhoea**	Mild or transient, 3-4 times/24 hours, with abnormal stool traits, or mild diarrhea lasting less than 1 week	Moderate or persistent, 5-7 times/24 hours, with abnormal stool traits, or diarrhea > 1 week	> 7 times/24 hours, abnormal stool traits, or hemorrhagic diarrhea, orthostatic hypotension, electrolyte imbalance, intravenous fluids required > 2 L	Hypotensive shock requires hospitalization
**Anorexia**	Decreased appetite, but no reduction in food intake	Appetite decreased, food intake decreased, but weight was not significantly reduced	Decreased appetite and significant weight loss	Need for interventions (e.g., gastric tube feeding, parenteral nutrition)
**Vomiting**	1~2 times/24 hours and daily activities not affected	3 ～ 5 times/24 hours or limited activity or persistent nausea leads to reduced food intake (24-48 hours)	Over 6 times within 24 hours or requiring intravenous infusion or persistent nausea leads to almost no food intake (>48 hours)	Hospitalization or other nutrition channels indicated due to hypotensive shock
**Nausea**	Transient (< 24 hours) or intermittent and food intake is normal	Persistent nausea leads to decreased food intake (24 to 48 hours)	Persistent nausea results in almost no food intake (>48 hours) or the need for intravenous fluids	Life-threatening (e.g., hypotensive shock)

	Grade 1	Grade 2	Grade 3	Grade 4
**Muscular pain (not at the inoculation site)**	Daily activities not affected	Daily activities marginally affected	Severe muscle pain, and daily activities severely affected	Urgent intervention or hospitalization indicated
**Headache**	Daily activities not affected, and treatment not required	Transient, daily activities marginally affected, treatment or intervention probably required	Daily activities severely affected, treatment or intervention required	Refractory, urgent intervention or hospitalization indicated
**Cough**	Transient, no treatment required	Persistent cough, effective treatment	Bouts of cough, uncontrollable treatment	Emergency or hospitalization
**Fatigue and weakness**	Hyperemia < 48 hours, no impact on activity	Hyperemia for 20%~50%>48 hours, with slight impact on activity	Hyperemia for >50%, with heavy impact on activity and stop working	Incapable of taking care of oneself, and urgent intervention or hospitalization indicated
**Fever (axillary temperature)**	37.3～＜38.0	38.0～＜38.5	38.5～＜39.5	≥39.5, lasting over 3 days

* Indicates type I hypersensitivity.

Adverse events not included in the above grading tables should be graded and evaluated according to the following criteria：

- Grade 1 Mild: Short-term (<48 hours) or slight discomfort, no effect on activities, treatment not required.
- Grade 2 Moderate: Mild or moderate restricted activities, presentation probably required, treatment not required or mild treatment required.
- Grade 3 Severe: Significant restricted activities, presentation and treatment required, hospitalization probably required.
- Grade 4 Critical: Life-threatening possibly, severely restricted activities, intensive care required.
- Grade 5 Death.

9.2 Outcomes of adverse events

The outcomes of adverse events include: Recovery; Not yet recovered; Recovered but sequelae; Death or Loss of visit.

9.3 Relationship between adverse events and Vaccination

Definitively unrelated: The subject has not used the investigational vaccine; or the adverse event occurs in an implausible time relationship to administration of the investigational vaccine; or there are other significant reasons that may result in the adverse event.

Unlikely related: There is evidence of administration of the investigational vaccine; the adverse event is more likely to be caused by other reasons; a negative or uncertain result is observed after re-administration of the investigational vaccine.

Possibly related: There is evidence of administration of the investigational vaccine; the adverse event occurs in a plausible time relationship to administration of the investigational vaccine; administration of the investigational vaccine cannot be ruled out as a cause of the adverse event, but other reasons may be the cause.

Probably related: There is evidence of administration of the investigational vaccine; the adverse event occurs in a plausible time relationship to administration of the investigational vaccine; the occurrence of the adverse events is explained by the investigational vaccine more reasonably than other reasons.

Definitely related: There is evidence of administration of the investigational vaccine; the adverse event occurs in a plausible time relationship to administration of the investigational vaccine; the occurrence of the adverse events is explained by the investigational vaccine more reasonably than other reasons; a positive result is observed after re-administration of the investigational vaccine; the adverse events are consistent with previous knowledge of this or this type of vaccine.

9.4 Documentation and Reporting of adverse events

All reportable adverse events that occur during the observation period set in this protocol will be reported by the investigator on the adverse events log of the CRF. Instructions for reporting adverse events are provided in the investigator's study file.

Adverse events that fulfill a reason for expedited reporting must be documented in the CRF within 24 hours and an email notification will be sent to a specified list of Trial Coordinating Centre representatives (including the medical monitor).

The investigator must also inform the study monitor in all cases. The initial report must be as complete as possible, including details of the current illness and (serious) adverse event, and an assessment of the causal relationship between the event and the study treatment. The Investigator will submit reportable adverse events to the relevant ethics committees in accordance with local ethics committee reporting requirements.
