# Supplementary material for: Retrospective study of the immunogenicity and safety of the CoronaVac SARS-CoV-2 vaccine in people with underlying medical conditions
Source: Commun Med (Lond). 2022 Nov 25;2:151. doi: 10.1038/s43856-022-00216-2 (PMC9700702; doi:10.1038/s43856-022-00216-2)
Supplement: Supplementary file 5 — Description of Additional Supplementary Files [file 43856_2022_216_MOESM5_ESM.pdf]

Description of Additional Supplementary Files

File Name: Supplementary Data 1

Description: The adverse events reported within 14 days post the first dose and the second dose of the vaccination 969 participants. The source data for main Figure 2.

File Name: Supplementary Data 2

Description: Titers of neutralizing antibodies to live SARS-CoV-2 virus (wild type) induced 14-28 days, 90 days, and 180 days after two-dose CoronaVac. The source data for main Figure 3.

File Name: Supplementary Data 3

Description: The SARS-CoV-2-spesific T cell responses after 3 months, and 6 months after two-dose CoronaVac, quantified by AIM assays. The source data for main Figure 4.
