# Supplementary material for: Retrospective study of the immunogenicity and safety of the CoronaVac SARS-CoV-2 vaccine in people with underlying medical conditions
Source: Commun Med (Lond). 2022 Nov 25;2:151. doi: 10.1038/s43856-022-00216-2 (PMC9700702; doi:10.1038/s43856-022-00216-2)
Supplement: Supplementary file 7 — Reporting Summary [file 43856_2022_216_MOESM7_ESM.pdf]

nature portfolio

Corresponding author(s): Zijie Zhang
Last updated by author(s): Nov 8, 2022

Reporting Summary

Nature Portfolio wishes to improve the reproducibility of the work that we publish. This form provides structure for consistency and transparency in reporting. For further information on Nature Portfolio policies, see our Editorial Policies and the Editorial Policy Checklist.

Statistics

For all statistical analyses, confirm that the following items are present in the figure legend, table legend, main text, or Methods section.

n/a	Confirmed	
☐	☒	The exact sample size (n) for each experimental group/condition, given as a discrete number and unit of measurement
☐	☒	A statement on whether measurements were taken from distinct samples or whether the same sample was measured repeatedly
☐	☒	The statistical test(s) used AND whether they are one- or two-sided<br>*Only common tests should be described solely by name; describe more complex techniques in the Methods section.*
☐	☒	A description of all covariates tested
☒	☐	A description of any assumptions or corrections, such as tests of normality and adjustment for multiple comparisons
☐	☒	A full description of the statistical parameters including central tendency (e.g. means) or other basic estimates (e.g. regression coefficient) AND variation (e.g. standard deviation) or associated estimates of uncertainty (e.g. confidence intervals)
☐	☒	For null hypothesis testing, the test statistic (e.g. F, t, r) with confidence intervals, effect sizes, degrees of freedom and P value noted<br>*Give P values as exact values whenever suitable.*
☒	☐	For Bayesian analysis, information on the choice of priors and Markov chain Monte Carlo settings
☒	☐	For hierarchical and complex designs, identification of the appropriate level for tests and full reporting of outcomes
☒	☐	Estimates of effect sizes (e.g. Cohen's d, Pearson's r), indicating how they were calculated

Our web collection on statistics for biologists contains articles on many of the points above.

Software and code

Policy information about availability of computer code

Data collection	R 4.2.0
Data analysis	R 4.2.0

For manuscripts utilizing custom algorithms or software that are central to the research but not yet described in published literature, software must be made available to editors and reviewers. We strongly encourage code deposition in a community repository (e.g. GitHub). See the Nature Portfolio guidelines for submitting code & software for further information.

Data

Policy information about availability of data

All manuscripts must include a data availability statement. This statement should provide the following information, where applicable:

- Accession codes, unique identifiers, or web links for publicly available datasets
- A description of any restrictions on data availability
- For clinical datasets or third party data, please ensure that the statement adheres to our policy

De-identified data are freely available upon request to the corresponding author.

Human research participants

Policy information about studies involving human research participants and Sex and Gender in Research.

Reporting on sex and gender	Sex was considered in our study, and sex of participants was determined based on self-report and confirmed by researchers at clinical sites.
Population characteristics	Baseline demographic characteristics (age,height, weight,BMI, sex and nationality) of the comorbidities group and healthy control were well-balanced across age groups.
Recruitment	We recruited participants who have received 2 doses of CoronaVac inactivated vaccine with 3-5 weeks of dose interval and were at the 14th-28th day after the second dose at the time of enrollment. Participants were eligible if they were 40 years of age or older, healthy, or diagnosed with any of the 6 most common chronic diseases: hypertension, diabetes mellitus (DM), coronary artery disease (CAD), chronic respiratory disease (CRD), obesity, and cancer, and were able to understand and complete the questionnaires. They will be excluded according to established criteria: had been infected by SARS-CoV-2; had received non-CoronaVac vaccine; with severe mental and neurological diseases; with any other factors unsuitable for clinical observation.Sex matched healthy participants were recruited as the control group.
Ethics oversight	Committee on Human Subject Research and Ethics of Yunnan University

Note that full information on the approval of the study protocol must also be provided in the manuscript.

Field-specific reporting

Please select the one below that is the best fit for your research. If you are not sure, read the appropriate sections before making your selection.

☒ Life sciences ☐ Behavioural & social sciences ☐ Ecological, evolutionary & environmental sciences

For a reference copy of the document with all sections, see nature.com/documents/nr-reporting-summary-flat.pdf

Life sciences study design

All studies must disclose on these points even when the disclosure is negative.

Sample size	The sample size was determined by considering statistical power and feasibility in practice. We aimed to recruit 200 individuals for diseases groups that are more common in the cities of study and 100 individuals for diseases that are less common the cities of study.
Data exclusions	Participant will be excluded according to established criteria: had been infected by SARS-CoV-2; had received non-CoronaVac vaccine; with severe mental and neurological diseases; with any other factors unsuitable for clinical observation; or they failed to be on site.
Replication	Our study design is cohort study, in which different individuals were considered as biological replicates. The significance of results was determined by statistical tests leveraging variation across different individuals (replications).
Randomization	Not relevant to our study, as this is a retrospective cohort study.
Blinding	Not relevant to our study, as this is a retrospective cohort study, without intervention.

Reporting for specific materials, systems and methods

We require information from authors about some types of materials, experimental systems and methods used in many studies. Here, indicate whether each material, system or method listed is relevant to your study. If you are not sure if a list item applies to your research, read the appropriate section before selecting a response.

Materials & experimental systems

n/a	Involved in the study	
☐	☒	Antibodies
☒	☐	Eukaryotic cell lines
☒	☐	Palaeontology and archaeology
☒	☐	Animals and other organisms
☐	☒	Clinical data
☒	☐	Dual use research of concern

Methods

n/a	Involved in the study	
☒	☐	ChIP-seq
☐	☒	Flow cytometry
☒	☐	MRI-based neuroimaging

Antibodies

Antibodies used	CD3-PerCP/Cyanine5.5,Biolegend,317336; CD4-FITC,Biolegend,357406; CD8-APC/Cyanine7,Invitrogen, 344714; CD69-PE/Dazzle, Biolegend, 310942; CD137-APC, Biolegend, 309810; CD134-PE/Cyanine7, Biolegend, 350012.
Validation	We validated all the antibodies by titration using the PBMCs.

Clinical data

Policy information about clinical studies
All manuscripts should comply with the ICMJE guidelines for publication of clinical research and a completed CONSORT checklist must be included with all submissions.

Clinical trial registration	This study is registered with ChiCTR.org.cn, ChiCTR2200058281.
Study protocol	The study protocol is provided as the supplementary information.
Data collection	Between Jul 5 and Dec 30, 2021, we recruited 1,302 participants in four different study sites (Haikou city, Wenchang city, and Qionghai city, Hainan Province; Kunming city, Yunnan Province; China).
Outcomes	The primary safety endpoint was the occurrence of adverse events within 14 days after the first dose and the second dose of the vaccination. The primary immunogenic endpoints were the seropositive rate and the geometric mean titers (GMTs) of neutralizing antibodies to live SARS-CoV-2 virus (wild type) 14-28 days, 90 days, and 180 days after two-dose vaccination. The secondary immunogenic endpoint was cellular responses (as measured by AIM assay) post 90 days and 180 days of two-dose vaccination.

Flow Cytometry

Plots

Confirm that:

- [x] The axis labels state the marker and fluorochrome used (e.g. CD4-FITC).
- [x] The axis scales are clearly visible. Include numbers along axes only for bottom left plot of group (a 'group' is an analysis of identical markers).
- [ ] All plots are contour plots with outliers or pseudocolor plots.
- [x] A numerical value for number of cells or percentage (with statistics) is provided.

Methodology

Sample preparation	PBMCs were cultured for 24 hours in the presence of SARS-CoV-2 specific MP in 96-wells U bottom plates in complete RPMI containing 5% Human AB Serum (Gemini Bioproducts). For the surface stain, to remove the cell culture medium, cells were washed with staining buffer (BD). Cells were then resuspended and stained with antibody cocktail for 30 minutes at RT in the dark, washed, and resuspended by staining buffer.
Instrument	Beckman DxFLEX
Software	FlowJo_v10.8.0
Cell population abundance	The SARS-CoV-2-specific CD4+ T cell responses were defined by CD3+, CD4+, OX40+ and CD137+. The SARS-CoV-2-specific CD8+ T cell responses (CD69+ CD137+) were defined by CD3+, CD8+, CD69+ and CD137+.
Gating strategy	We gated the lymphocyte by the FSC/SSC ;then gated the single cell by the FSC/SSC; gated CD3+ for T cells; gated CD4+, OX40 + and CD137+ for SARS-CoV-2 specific CD4 T cells; gated CD8+, CD69+ and CD137+for SARS-CoV-2 specific CD8 T cells;

- [x] Tick this box to confirm that a figure exemplifying the gating strategy is provided in the Supplementary Information.
